# ATGL links insulin dysregulation to insulin resistance in adolescents with obesity and hepatosteatosis

**DOI:** 10.1172/JCI184740

**Published:** 2025-03-17

**Authors:** Aaron L. Slusher, Nicola Santoro, Alla Vash-Margita, Alfonso Galderisi, Pamela Hu, Fuyuze Tokoglu, Zhongyao Li, Elena Tarabra, Jordan Strober, Daniel F. Vatner, Gerald I. Shulman, Sonia Caprio

**Affiliations:** 1 Department of Pediatrics,; 2 Department of Obstetrics, Gynecology and Reproductive Sciences, and; 3 Radiology and Biomedical Imaging, Yale School of Medicine, New Haven, Connecticut, USA.; 4 Alexion Pharmaceuticals Inc., New Haven, Connecticut, USA.; 5 Department of Internal Medicine and; 6 Department of Cellular and Molecular Physiology, Yale School of Medicine, New Haven, Connecticut, USA.

**Keywords:** Endocrinology, Metabolism, Adipose tissue, Diabetes, Obesity

## Abstract

**BACKGROUND:**

This study examined the underlying cellular mechanisms associated with insulin resistance (IR) and metabolic disease risk within subcutaneous adipose tissue (SAT) in youth with obesity and IR compared with those without IR.

**METHODS:**

Thirteen adolescents who were insulin sensitive (IS) and 17 adolescents with IR and obesity underwent a 3-hour oral glucose tolerance test and MRI to measure abdominal fat distribution and liver fat content. Lipolysis was determined by glycerol turnover ([^2^H5]-glycerol infusion) and adipose triglyceride lipase (ATGL) phosphorylation (Western blot) from SAT samples biopsied prior to and 30-minutes following insulin infusion during a hyperinsulinemic-euglycemic clamp (HEC).

**RESULTS:**

Glycerol turnover suppression during the HEC (first step) was lower in participants with IR compared with those with IS. Prior to insulin infusion, activated ATGL (reflected by the p-ATGL (Ser^406^)-to-ATGL ratio) was greater in participants with IR compared with those with IS and suppressed in response to a 30-minute insulin exposure in participants with IS, but not in those with IR. Lastly, greater ATGL inactivation is associated with greater glycerol suppression and lower liver fat.

**CONCLUSIONS:**

Insulin-mediated inhibition of adipose tissue lipolysis via ATGL is dysregulated among adolescents with IR compared with those with IS, thereby serving as a vital mechanism linking glucose and insulin dysregulation and ectopic lipid storage within the liver.

**FUNDING:**

This work was supported by funding from the NIH (R01-HD028016-25A1, T32- DK-007058, R01-DK124272, RO1-DK119968, R01MD015974, RO1-DK113984, P3-DK045735, RO1-DK133143, and RC2-DK120534) and the Robert E. Leet and Clara Guthrie Patterson Trust Mentored Research Award.

## Abstract 2

Hormone Sensitive Lipase; Metabolic dysfunction-associated steatotic liver disease; Proton Density Fat Fraction; Subcutaneous Adipose Tissue

## Introduction

Pediatric obesity has increased nearly 4-fold in the United States over the past 5 decades (1, 2). The rising prevalence of obesity-related insulin resistance (IR) in adolescents is linked to rising rates of Type 2 diabetes (T2D), metabolic dysfunction-associated steatotic liver disease (MASLD; refs. 3–6), and the increased risk of death in early adulthood from cardiometabolic and liver diseases (7, 8). However, little is known about the underlying cellular and molecular mechanisms associated with increased insulin resistance and the concomitant metabolic disease risk observed in adolescents with obesity.

Whole body insulin resistance is accompanied by an excess output of nonesterified fatty acids (NEFA) from the adipose into the circulation and their consequent accumulation in ectopic tissues such as the muscle and liver (9). Only recently have the molecular mechanisms driving adipose tissue IR (AT-IR) in youth with obesity begun to be elucidated (10), yet research focusing on molecular mechanisms is largely limited to animal studies. For example, Lyu et al. (11, 12) recently detailed the mechanism of white AT-IR within various rodent models undergoing short-term overnutrition, suggesting that when IR occurs, insulin inhibition of key lipolytic enzymes (such as adipose triglyceride lipase [ATGL]) is impaired. Therefore, the present study aims to examine, for what is, to our knowledge, the first time, the intracellular mechanisms of insulin-mediated signaling within subcutaneous adipose tissue (SAT) in youth with obesity and IR compared with those less severe IR (hereby referred to as insulin sensitive [IS]). In this study, Insulin-mediated suppression of adipose tissue lipolysis, reflected by glycerol turnover, was assessed by isotope dilution during a [^2^H5]-glycerol tracer infusion. Insulin-mediated suppression of ATGL (Ser^406^) phosphorylation was assessed in SAT during a hyperinsulinemic-euglycemic clamp (HEC). Additionally, the participants underwent a 3-hour oral glucose tolerance test (OGTT) and multisection MRI to measure abdominal fat distribution and liver fat content.

## Results

### Descriptive, anthropometric, and metabolic characteristics of participants.

Descriptive characteristics of the participants are shown in Table 1. In brief, no differences were observed for age or body anthropometrics, including height, weight, BMI, body fat percentage, or fat-free body mass. Although no differences in waist and hip circumferences or SAT and associated subsections (deep, superficial, and the ratio of deep-to-superficial SAT) were observed between groups, VAT and the ratio of VAT-to-total (VAT+SAT) were greater in participants with IR compared with those with IS (*t*_[25]_ = –3.385, *P* = 0.002; *t*_[25]_ = –4.050, *P* < 0.001, respectively). Of note, hepatic fat content, expressed as proton density fat fraction (PDFF), was greater in participants with IR compared with those with IS (*U* = 29.00, *P* = 0.002). The ratio between leg fat and total fat measured by DEXA was higher in youth with insulin sensitivity (0.19 ± 0.016) than in those showing insulin resistance (0.17 ± 0.019) (*P* = 0.001).

Fasting glucose concentrations were similar between groups, whereas fasting insulin and C-peptide concentrations were greater in participants with IR compared with those with IS (*t*_[18.371]_ = –5.605, *P* < 0.001; *t*_[19.658]_ = –4.853, *P* < 0.001, respectively). In response to the 3-hour OGTT, the glucose (*F*_[2.858,__71.455]_ = 2.864, *P* = 0.045), insulin (*F*_[2.892,__72.292]_ = 5.423, *P* = 0.002), and C-peptide (*F*_[3.404,__81.697]_ = 4.691, *P* = 0.003) responses were greater in participants with IR compared with those with IS (Supplemental Figure 1 A–C; supplemental material available online with this article; https://doi.org/10.1172/JCI184740DS1). As a result, whole-body insulin sensitivity index (WBISI) was lower, and homeostatic model assessment for insulin resistance (HOMA-IR) was greater in participants with IR compared with those with IS (*U* = 7.00, *P* < 0.001; *U* = 6.00, *P* < 0.001, respectively; Table 1).

### Hyperinsulinemic-euglycemic clamp.

**Figure 1:**
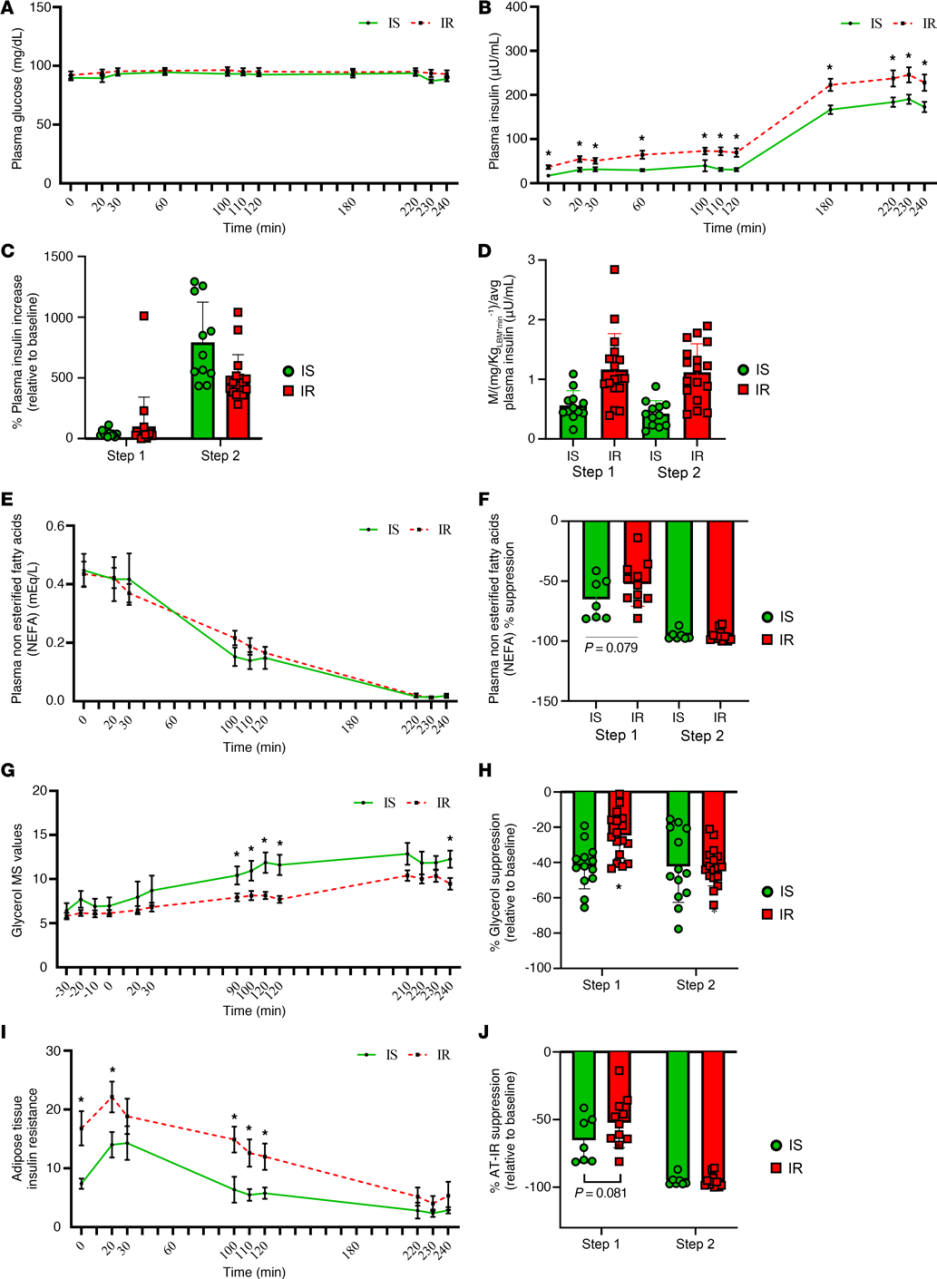
In response to the HEC, no differences in plasma glucose were observed between groups. (**A**) Plasma insulin concentrations were greater in participants with IR compared with those with IS throughout the HEC, whereas relative increases in plasma insulin concentrations (compared with time point 0) were similar in both groups (**B** and **C**). Peripheral insulin sensitivity (M; adjusted for plasma insulin concentrations) during the second step of the HEC was lower in participants with IR compared with those with IS (**D**). Insulin-induced suppression of plasma non esterified fatty acid (NEFA) concentrations tended to be lower in participants with IR compared with those with IS during the first step of the HEC and were suppressed similarly in both groups throughout the HEC (**E** and **F**). Glycerol turnover rates reached steady state during each step of the HEC in both participant groups, and glycerol turnover rates were lower in participants with IR compared with those with IS throughout step 1 of the HEC (**G**). Consistent with these findings, insulin-induced suppression of glycerol turnover during the first step of the HEC was lower in participants with IR compared with those with IS (**H**). Finally, AT-IR was elevated in participants with IR compared with those with IS at the initiation of HEC and remained elevated during the first step. Similarly, the level of suppression tended to be lower in participants with IR compared with those with IS before near complete suppression was observed in both participant groups (**I** and **J**). Differences in continuous data were determined by Student’s *t* test or Mann–Whitney U analysis, and the impact of time on plasma protein and indices of insulin resistance in response to HEC tests were examined by repeated measures ANOVA. *P* values less than 0.05 were considered significant. Data are presented as means ± SD or IQR (25%, 75%). *P > 0.05.

**Figure 2:**
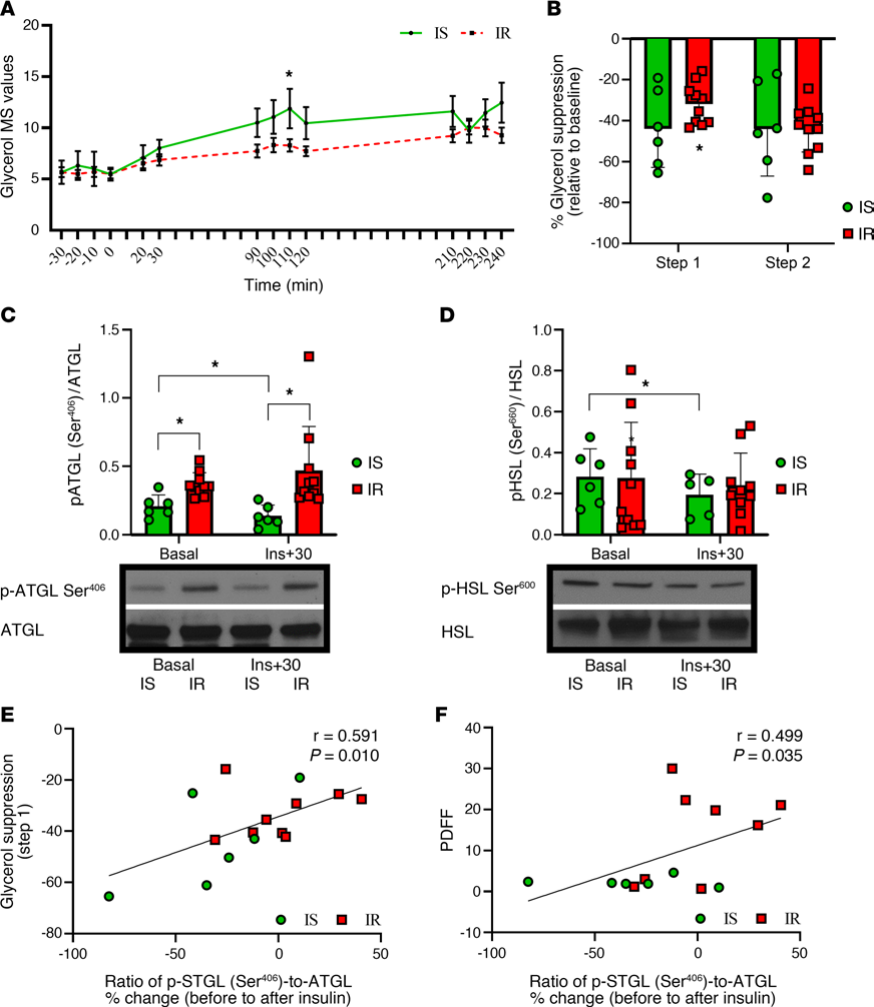
Glycerol turnover rates reached steady state during each step of the HEC in both participant groups, and glycerol turnover rates were lower in participants with IR compared with those with IS throughout step 1 of the HEC. (**A**) Consistent with these findings, insulin-induced suppression of glycerol turnover during the first step of the HEC was lower in participants with IR compared with those with IS (**B**). Representative Western blot images for the ratios of p-ATGL (Ser^406^)-to-ATGL and p-HSL (Ser^660^)-to-HSL prior to and in response to acute, low-dose insulin infusion (30 minutes following the initiation of 8 mU·m^–2^·min^–1^ insulin). Prior to insulin, the ratio of p-ATGL (Ser^406^)-to-ATGL was greater in IR compared with IS participants, whereas a decrease in the ratios of p-ATGL (Ser^406^)-to-ATGL was observed in participants with IS, but not IR, following insulin infusion (**C**). Lastly, no differences in the ratio of p-HSL (Ser^660^)-to-HSL protein were observed between IS and IR participant groups prior to insulin. However, p-HSL (Ser^660^)-to-HSL decreased in participants with IS, but not those with IR, following insulin infusion (**D**). Lastly, the reduced activation of the ratio of p-ATGL (Ser^406^)-to-ATGL, indicated by the percent change (before the start to 30 minutes after the start of insulin infusion), was associated with greater suppression glycerol (step 1; **E**) and lower PDFF (**F**). Differences in continuous data were determined by Student *t* test or Mann–Whitney U analysis and the impact of time on plasma protein and indices of insulin resistance in response to HEC tests were examined by 2-way repeated measures ANOVA. Additionally, differences in the phosphorylation ratios were compared by using a 2-way repeated measures ANOVA and Pearson’s correlations were used to examine the relationship of ATGL protein analysis with indices of insulin resistance. *P* values less than 0.05 were considered significant. Data are presented as means ± SD or IQR (25%, 75%). *P > 0.05.

During the clamp infusion, plasma glucose concentrations were similar among participants with IR and IS (*F*_[4.517, 121.967]_ = 0.647, *P* = 0.648; Figure 1A). Plasma insulin concentrations were greater in participants with IR compared with those with IS throughout the HEC, whereas relative increases in plasma insulin concentrations (compared to time point 0) were similar in both groups (*F*_[2.415,__26.568]_ = 1.352, *P* = 0.278; *F*_[1,__15]_ = 2.861, *P* = 0.111, respectively; Figures 1, B and C). Peripheral insulin sensitivity (M; adjusted for plasma insulin concentrations) during the second step of the HEC was lower in participants with IR compared with those with IS (*P* < 0.001; Figure 1D). In addition, insulin-induced suppression of plasma nonesterified fatty acids (NEFA) concentrations tended to be lower in participants with IR compared with those with IS during the first step of the HEC (*P* = 0.079) and were suppressed similarly in both groups throughout the HEC (*F*_[1.869,__28.033]_ = 0.878, *P* = 0.537; *F*_[1,__16]_ = 1.981, *P* = 0.178, respectively; Figure 1, E and F). Although glycerol turnover rates reached steady state during each step of the HEC in both groups, glycerol turnover rates were lower in participants with IR compared with those with IS throughout step 1 of the HEC (*F*_[4.718,__70.765]_ = 0.868, *P* = 0.502; Figure 1G), and insulin-induced suppression of glycerol turnover during the first step of the HEC was lower in participants with IR compared with those with IS (*P* = 0.022; Figures 2H). No differences in plasma glycerol were observed between participants with IR and IS (Supplemental Figure 2). Lastly, AT-IR was elevated in participants with IR compared with those with IS at the initiation of HEC and remained elevated during the first step, and the level of suppression tended to be lower in participants with IR compared with those with IS (*P* = 0.081), before near complete suppression was observed in both participant groups (*F*_[2.225,__22.253]_ = 1.139, *P* = 0.343; Figure 1, I and J).

### Lipolytic Pathway Analysis.

Sixteen participants (*n* (IS) = 6; *n* (IR) = 10) agreed to provide abdominal adipose tissue biopsies prior to and 30 minutes following the initiation of insulin infusion during the first step of the HEC. The descriptive characteristics, OGTT data, and HEC data for this subset of participants can be examined in Table 2 and are similar to the cohort described above. Glycerol turnover rates reached steady state during each step of the HEC in both groups (*F*_[3.787, 49.229]_ = 1.007, *P* = 0.410; Figure 2A), and insulin-induced suppression of glycerol turnover during the first step of the HEC was lower in participants with IR compared with those with IS (*P* = 0.048; Figure 2B). In addition, a group effect was observed for the ratio of p-ATGL (Ser^406^)-to-ATGL (*P* = 0.006). More specifically, the ratio of p-ATGL (Ser^406^)-to-ATGL was greater in participants with IR compared with those with IS prior to insulin infusion (*P* = 0.001; Figure 2C), whereas no differences were observed for the ratio of hormone-sensitive lipase (HSL [Ser^660^])-to-HSL protein between IS and IR participant groups (*P* = 0.478; Figure 2D). In response to acute insulin exposure (30-minutes following the initiation of 8 mU/m^2^/min), and decreases in the ratios of p-ATGL (Ser^406^)-to-ATGL and p-HSL (Ser^660^)-to-HSL were observed in participants with IS, but not IR (*P* = 0.044; *P* = 0.010; Figure 2, C and D). The changes in phosphorylation ratios were compared by using a 2-way ANOVA and the group effect on changes of ATGL phosphorylation between the group was still statistically significant (*P* = 0.006). The interaction term (before/after insulin and IR/IS status) was not significant (*P* = 0.216), this supporting a difference in the ratios of p-ATGL between IR and IS both at baseline and after insulin suppression. Interestingly, the reduction in the ratio of p-ATGL (Ser^406^)-to-ATGL in response to acute insulin exposure (determined by the percent change relative to preinsulin infusion) was associated with greater suppression of glycerol (step 1; r = 0.591, *P* = 0.010; Figure 2E) and lower PDFF (r = 0.499, *P* = 0.035; Figure 2F). No other associations were observed between insulin-mediated changes to ATGL or HSL phosphorylation with indices of insulin resistance.

## Discussion

This study combined highly sensitive metabolic profiling (OGTT and HEC) and abdominal and hepatic fat patterning (MRI coupled with PDFF) with analysis of the intracellular lipolytic pathway from biopsied SAT obtained prior to and following short-term exposure to insulin in vivo. The primary findings demonstrate that short-term exposure of abdominal adipose tissue to insulin (8 mU/m^2^/min) does not dephosphorylate ATGL and HSL in participants with IR compared with those with IS. This resistance of adipose tissue to suppress lipolysis in response to insulin is reflected by elevated indices of AT-IR and less suppressed glycerol during the first step of the HEC. As a result, these findings bring to light an important mechanism by which AT-IR contributes to the failure of abdominal SAT to retain and store lipids that potentially accumulate ectopically within the liver.

Research in youth with obesity is critically important to identify potential therapeutic targets and early intervention strategies that mitigate the deleterious health outcomes that these youth may experience as they mature into adulthood. Differences among individuals who are metabolically healthy compared with those who are unhealthy and have obesity provides an opportunity to identify a potential mechanism to achieve these goals, yet previous research in adolescent populations is often limited to noninvasive techniques to investigate pathways of metabolic dysfunction or assessments of the molecular pathways at a single time point under fasting conditions. For example, our group previously showed that total CGI-58, a molecule involved in the regulation of lipolysis, was increased in adolescents with obesity and IR compared with those with IS (10). As an extension of that work, results from the present study demonstrate that the ratio of phosphorylated-to-total ATGL and HSL both decreased in the IS, but not the IR participant group in response to insulin stimulation. These findings provide support for the adverse expression of proteins involved in the regulation of the lipolytic pathway among participants with IR compared with those with IS, and the ability to examine changes in the ratios of phosphorylation-to-total protein prior to and in response to insulin infusion during the HEC enables deeper insight into targeting potential mechanisms of IR with adipose tissue. Thus, the marked difference in ATGL expression and responsiveness to insulin observed in participants with IS compared with those with IR supports its role as a potential rate limiting factor for adipose tissue lipolysis and highlights its potential as a key target for future interventions.

Overall, these findings also demonstrate that adipose tissue IR is an important component of whole-body IR in adolescents with obesity. Furthermore, the correlation between ATGL dephosphorylation and adipose tissue insulin sensitivity suggests that ATGL dysregulation is central to the development of IS in adolescents. Similarly, the preservation of some degree of insulin sensitivity within a subpopulation of adolescent children with obesity is worth additional investigation, and there is increasing recognition that these individuals may be resistant to the adverse metabolic effects of excess adiposity (13). Recently, Petersen et al. (14) characterized the physiological differences between metabolically healthy and metabolically unhealthy adults with obesity. Much like the population of adolescents with obesity examined in the present investigation, differences in metabolic profiles were associated with altered patterns of body fat distribution at any given BMI. In addition, abdominal adipose tissue gene expression in the adult study was consistent with findings from the present study: adipose tissue inflammation and extracellular matrix remodeling genes were higher and lipogenesis genes were lower in participants with obesity and with IR compared with those with IS (10, 15, 16).

This investigation is not without limitations. For example, the adolescent children under investigation are in a dynamic state of growth and are already classified as obese. Likewise, this study lacks a lean control population for additional comparisons between IS adolescents with and without obesity. Therefore, it is difficult to determine whether the IS subset should be considered metabolically healthy or a population that is in a state of flux and progressing toward an unhealthy phenotype, as previously demonstrated in adult populations (14). Nonetheless, despite the small sample size and lack of lean control population, these results still provide insight into potential mechanisms that delay the onset of severe insulin resistance and may allow for targeted interventions to halt or reverse the continuation of metabolic dysregulation as these children enter early adulthood. Lastly, this study was limited to SAT and did not include VAT biopsies, since it would have been unethical to biopsy the VAT depot in the otherwise healthy cohort of obese adolescents under the current protocol. Therefore, this study is limited to obtaining adipose tissue samples from the SAT depot and prevents comparing the mechanisms associated with insulin-mediated suppression of lipolysis that contribute to IR and hepatic fat content between the 2 adipose tissue depots.

In conclusion, this study demonstrates that the capacity for insulin to inhibit adipose tissue lipolysis via ATGL and HSL is dysregulated among adolescent children with IR compared with those with IS. These findings serve as a vital mechanism linking dysregulated glucose and insulin kinetics with the spillover of NEFAs that are stored ectopically within the liver. Furthermore, this excess accumulation of fat within the liver serves as an initial indication of liver disease progression that has been shown to manifest within adolescent children at an increased rate since the Sars-CoV-2 global pandemic (17). Likewise, considering recent findings demonstrating the potential for short-term dietary manipulation and physical activity engagement to positively regulate dynamics of adipose tissue lipolysis (12, 18, 19), these findings support the urgent need to develop effective lifestyle and pharmacological interventions to reverse the pathophysiological progression of insulin resistance prior to the manifestation of more severe metabolic disease during childhood and early adulthood.

## Methods

### Sex as a biological variable.

Male and female participants were recruited from the Yale Study of the Pathophysiology of Prediabetes/T2D in Youth, an ongoing investigation based at the Yale Pediatric Obesity Clinic aiming to identify the underlying pathophysiology of prediabetes in youth (NCT01967849). Similar findings are reported for both sexes.

### Study participants.

Recruited participants were characterized as being IS (WBISI ≥ 2.0) or IR (WBISI < 2.0) based on results from a 3-hour OGTT (described below). All IS and IR participants were classified as pubescent adolescents with obesity (BMI > 95^th^ percentile) aged between 12 and 21 years old and presented with a 2-hour glucose concentration < 200 mg/dL assessed during their previously administered OGTT. To control for the potential impact of the severity of BMI, all participants with IS and IR were matched as best as possible for age and BMI. Upon recruitment, all participants provided their written and informed consent and subsequently participated in a dual-energy x-ray absorptiometry (DEXA) scan to assess whole-body composition and the regional distribution of body fat with a Hologic scanner by a trained technician at the Yale Bone Center. In addition, complete medical history and physical examination was conducted upon recruitment at the Yale Center of Clinical Investigation (YCCI). Recruited participants were ineligible to participate in the investigations if they presented with elevated baseline creatine concentrations of greater-than 1.0 mg/dL, low hematocrit of less-than 35%, pregnancy, or previously diagnosed T2D with fasting plasma glucose of at least 126 mg/dl and/or hemoglobin A1c of at least 6.5%, endocrinopathies, cardiac, pulmonary, and/or other chronic illnesses. Likewise, participants with a current psychiatric disorder or use of anorexic agents were also ineligible to participate.

### OGTT.

All participants underwent a 3-hour OGTT performed at the YCCI. Whole blood samples were collected from an intravenous line inserted in the antecubital vein for the analysis of glucose, insulin, C-peptide, and glycerol at baseline, 10, 20, and 30 minutes, then every 30 minutes up to 180 minutes following oral glucose challenge (1.75 g/kg body weight: 75 g maximum). HOMA-IR and WBISI were calculated as previously described (20, 21). Finally, AT-IR was calculated by multiplying fasting insulin and fasting free fatty acid concentrations.

### Abdominal fat distribution and intrahepatic fat content by MRI-proton density fat fraction.

On the same day of the OGTT, a multisection abdominal MRI was performed at the Magnetic Resonance Research Center (Siemens Sonata 3.0 Tesla System; Erlangen, Germany). Abdominal SAT and VAT distribution patterns were determined using a threshold to discriminate fat from soft tissue at the level of the L4/L5 disc space on a single slice. Deep (DeepSAT) and superficial SAT (SupSAT) were determined based on their division by the fascia superficialis (22). The ratios of VAT-to-total (VAT+SAT) and DeepSAT-to-SupSAT were also calculated. Liver MRI has advanced from measuring the hepatic fat fraction to the more precise PDFF (23). PDFF provides a more accurate and precise estimation of liver fat content and is considered the unique quantitative imaging biomarker for hepatic steatosis currently available (24). For our study, MASLD was defined as a PDFF greater-than or equal-to 5.5 (25).

### Hyperinsulinemic-euglycemic clamp.

Following an overnight fast and a week following the DEXA scan, participants arrived at the YCCI where height, weight, and waist and hip circumferences were immediately measured. An IV line was inserted into each arm for blood sampling and the continuous infusion of [^2^H5]-glycerol at a concentration based on body surface area. The protocol was initiated by a continuous infusion of [^2^H5]-glycerol for a 2-hour period (10). Immediately thereafter, a first step insulin infusion (8 mU/m^2^/min) was initiated for a 2-hour period followed by a second step insulin infusion (80 mU/m^2^/min) for an additional 2 hours (26). Plasma samples were obtained at baseline, 30, 20, and 10 minutes, and immediately prior to the start of the first step insulin infusion. Additional plasma samples were obtained at various intervals during the HEC, including the final 10, 20, and 30 minutes of each insulin step, to examine the effects of insulin on circulating indices of metabolism as described below. Blood for [^2^H5]-glycerol enrichment was collected and plasma was immediately separated by centrifugation for storage at –70°C until analysis. Glycerol in plasma and infusates were analyzed by gas chromatography and mass spectrometry as previously described (27). All enrichment measurements were corrected for background enrichments.

### Subcutaneous adipose tissue biopsies.

Two abdominal SAT biopsies were performed after having applied EMLA cream and local anesthesia (lidocaine without epinephrine) under sterile conditions at the YCCI Hospital Research Unit. A 2-cm scalpel incision (approximately 5 cm from the umbilicus) was made for the removal of 2–3 g of abdominal SAT immediately prior to the start of insulin infusion during the HEC. To examine the sensitivity of adipose tissue to insulin, a second biopsy was obtained from the participant’s right side (approximately 5 cm from the umbilicus) 30 minutes after the start of the first step insulin infusion. A 3 g SAT sample was obtained from each biopsy.

### Biochemical analysis.

Glucose concentrations obtained during the OGTT and HEC were examined by bedside using a YSI2700-STAT–analyzer (Yellow Springs Instruments). Whole blood samples obtained during the OGTT and HEC were centrifuged for 20 minutes at 313*g* for the isolation of plasma and subsequent analysis of insulin (Linco) and nonesterified fatty acid (Fujifilm Wako Chemicals) concentrations at baseline and various time points throughout each protocol. Fatty acid concentrations were examined in 7 participants with IS and 11 with IR. The product of plasma fasting insulin and fasting nonesterified fatty acids were used to calculate an index of AT-IR.

### Western blotting.

A 250 mg flash-frozen SAT sample was utilized to examine insulin-mediated suppression of SAT lipolysis (ratio of phosphorylated to ATGL pSer^406^/ATGL and HSL pSer^600^/HSL). Total protein lysates were generated using T-per tissue protein extraction reagent containing Halt protease and phosphatase inhibitor cocktail (Thermo Fisher Scientific), and total protein concentrations were determined using Pierce 660 nm Protein Assay Kit (Thermo Fisher Scientific). Protein samples were prepared using 4× Laemmli buffer with β-mercaptoethenol (Bio-Rad Laboratories) and boiled for 5 minutes at 95°C. Fifteen micrograms of protein lysate were resolved by SDS-polyacrylamide gel electrophoresis, transferred to polyvinylidene fluoride membranes, and total protein loading was determined with ponceau stain (Sigma Aldrich). Membranes were subsequently destained in 0.1 M NaOH, washed in PBS with Tween, incubated for 1 hour in 5% nonfat dry milk, then incubated overnight at 4°C with a rabbit polyclonal antibody diluted to 1:2,000 in 5% nonfat dry milk; Insulin receptor (Cat. No. 3025; Cell Signaling Technology), Akt pSer^473^ (Cat. No. 4060S), Akt (Cat. No. 4691S), ATGL (Cat. No. 2138S), and HSL pSer^600^ (Cat. No. 45804S), HSL (Cat. No. 4107S), and ATGL pSer^406^ (Cat. No. ab135093-100ug; Abcam) were the primary antibodies used. The following morning, membranes were washed in PBS with Tween, incubated for 1 hour with anti-rabbit IgG horseradish peroxidase–linked secondary antibody diluted to 1:10,000 in 5% nonfat milk (Cat. No. 7074S; Cell Signaling Technology). Signals were detected in SuperSignal West Femto Maximum Sensitivity Substrate or West Pico PLUS Chemiluminescent Substrate (Thermo Fisher Scientific). Films were developed within the linear dynamic range of signal and scanned for digital analysis. Protein expression was normalized to total protein using ImageJ.

### Statistics.

Categorical variables were compared between groups using Pearson χ^2^ test. Differences in continuous data were determined by Student *t* test or Mann–Whitney U analysis. Additionally, the impact of time on plasma protein or cellular protein analysis in response to OGTT or HEC tests were examined by repeated measures ANOVA. Pearson’s correlations were used to examine the relationship of ATGL protein analysis with indices of insulin resistance. Hypothesis tests were conducted using an α of 0.05, and inference was supplemented by the magnitude of the estimated effect sizes. A *P* value of less than 0.05 was considered statistically significant.

### Study approval.

This study was approved by The Pediatric Yale University’s Institutional Review Board.

### Data Availability.

Deidentified data are available in the Supplemental Data Values file.

## Author contributions

SC directed the study. NS and GIS provided advice. ALS, ET, and SC conceived and planned the experiments. ALS, NS, AVM, PH, FT, ET, JS, and ZL performed the experiments. DFV and GIS provided technical advice. ALS, NS, AG, GIS, and SC contributed to the data analysis and interpretation of the results. ALS prepared the figures and the first draft of the manuscript. ALS, NS, and SC revised and finalized the manuscript.

## Supplementary Material

Supplemental data

ICMJE disclosure forms

Unedited blot and gel images

Supporting data values

## Figures and Tables

**Table 2:**
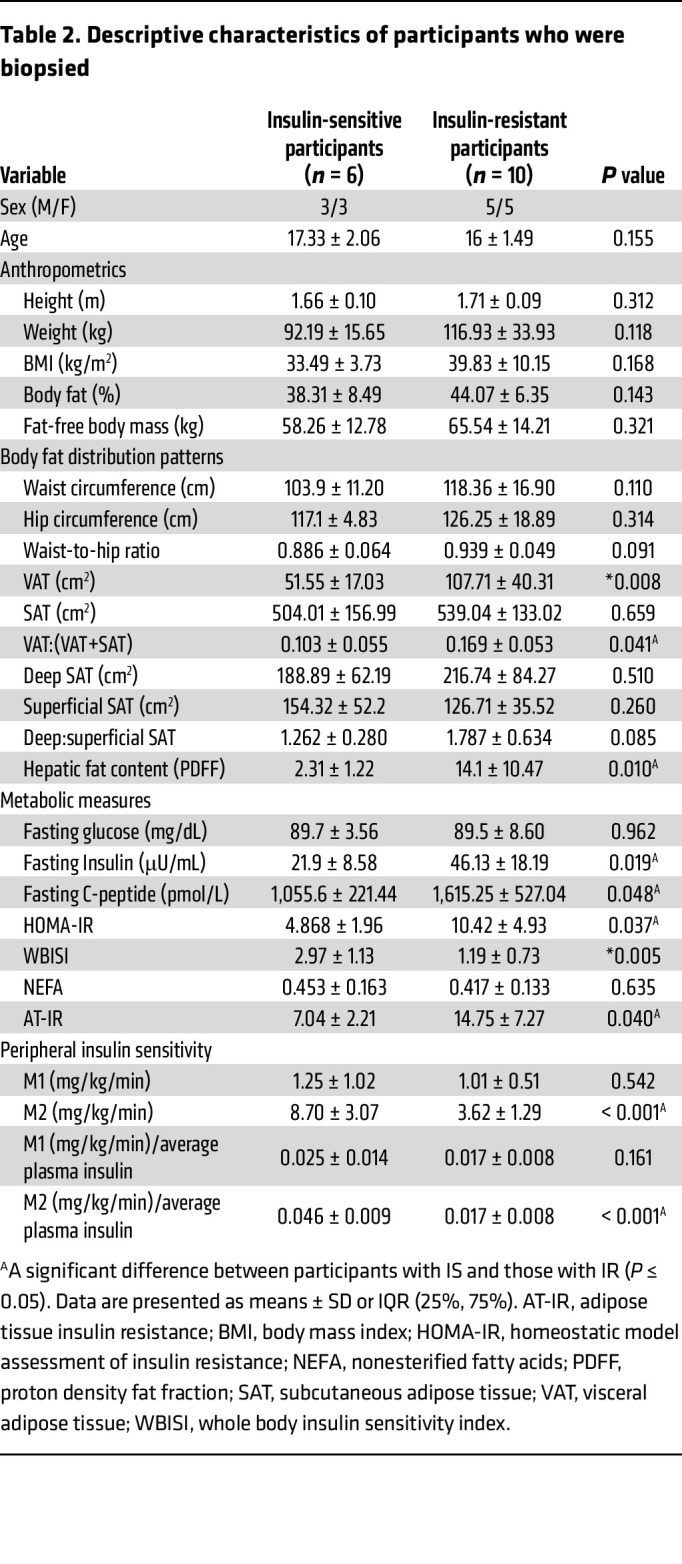
Descriptive characteristics of participants who were biopsied

**Table 1:**
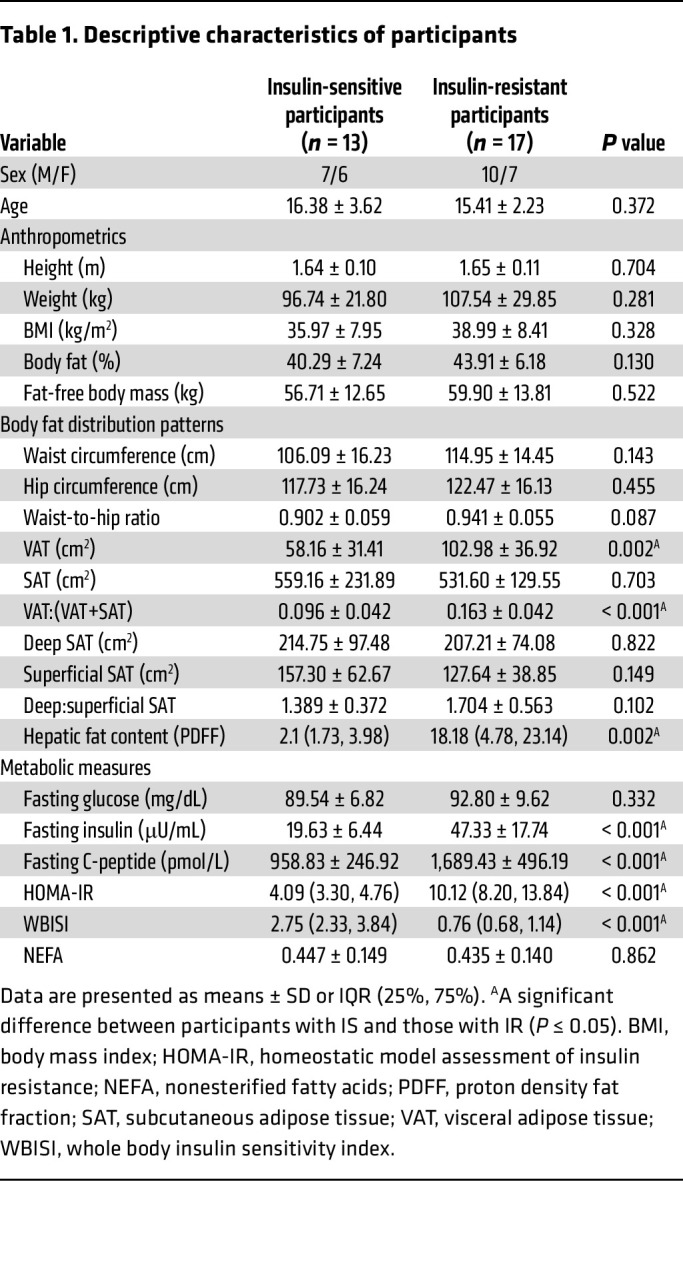
Descriptive characteristics of participants

## Version 1 03/17/2025

Electronic publication
