# Supplementary material for: ATGL links insulin dysregulation to insulin resistance in adolescents with obesity and hepatosteatosis
Source: J Clin Invest. 2025 Mar 17;135(6):e184740. doi: 10.1172/JCI184740 (PMC11910223; doi:10.1172/JCI184740)
Supplement: Supplemental data [file jci-135-184740-s100.pdf]

Supplemental Figure 1

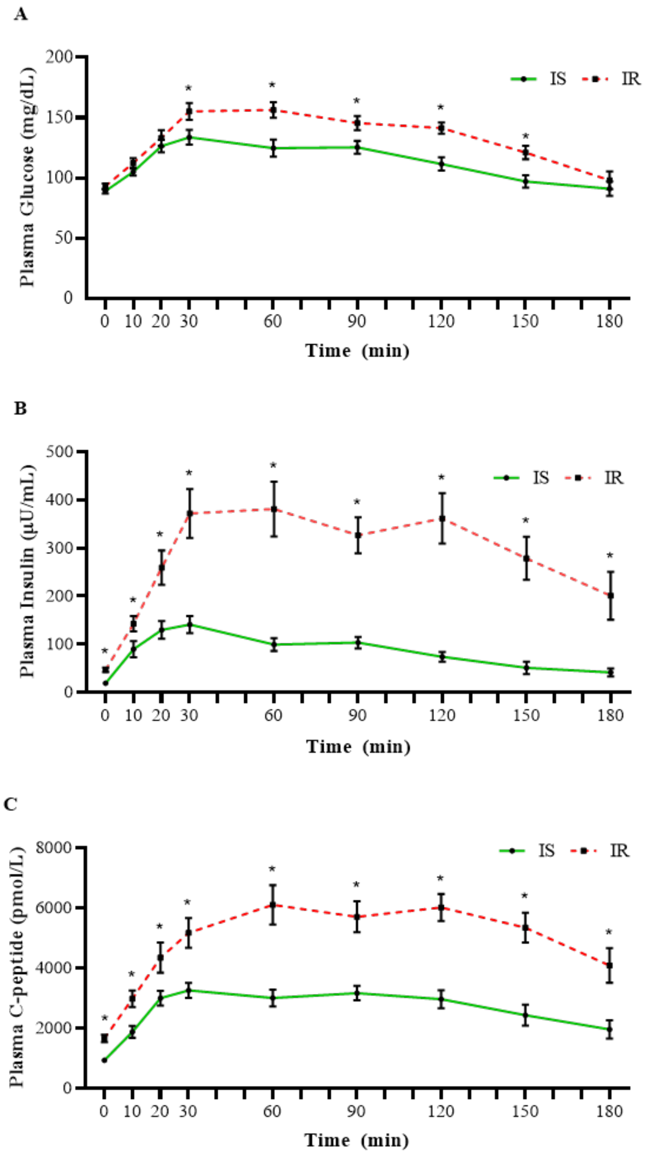

Supplemental Figure 1. Plasma glucose (panel A), insulin (panel B), and C-peptide (panel C) responses to the 3-hour OGTT were significantly greater in IR compared to IS subjects. Data are presented as means ± S.D.. The * indicates a significant difference in the IR relative to IS subject group at each respective time point.

Supplemental Figure 2

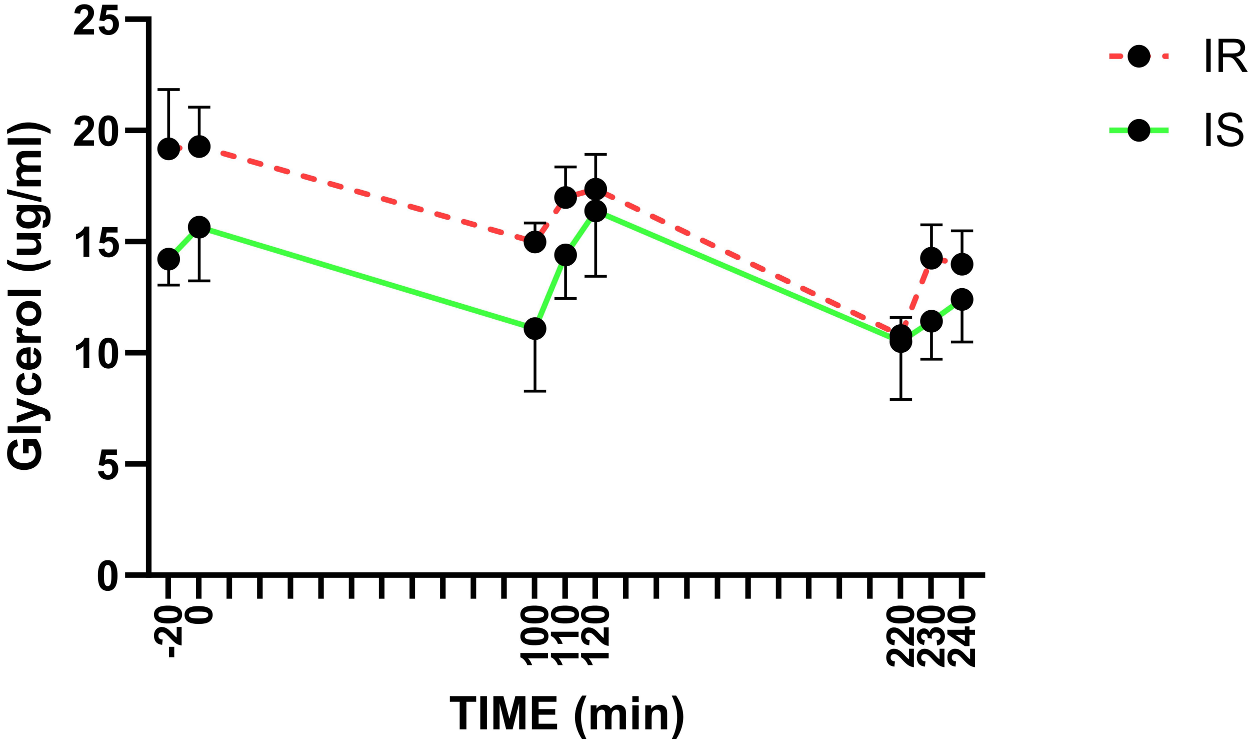

Supplemental figure 2. The figure shows plasma glycerol concentrations during the different phases of the HEC. There was no difference between the two groups in terms of plasma glycerol during the study.
