# Supplementary material for: ATGL links insulin dysregulation to insulin resistance in adolescents with obesity and hepatosteatosis
Source: J Clin Invest. 2025 Mar 17;135(6):e184740. doi: 10.1172/JCI184740 (PMC11910223; doi:10.1172/JCI184740)
Supplement: Unedited blot and gel images [file jci-135-184740-s102.pdf]

Figure 2

C

p-ATGL Ser^{406}

ATGL

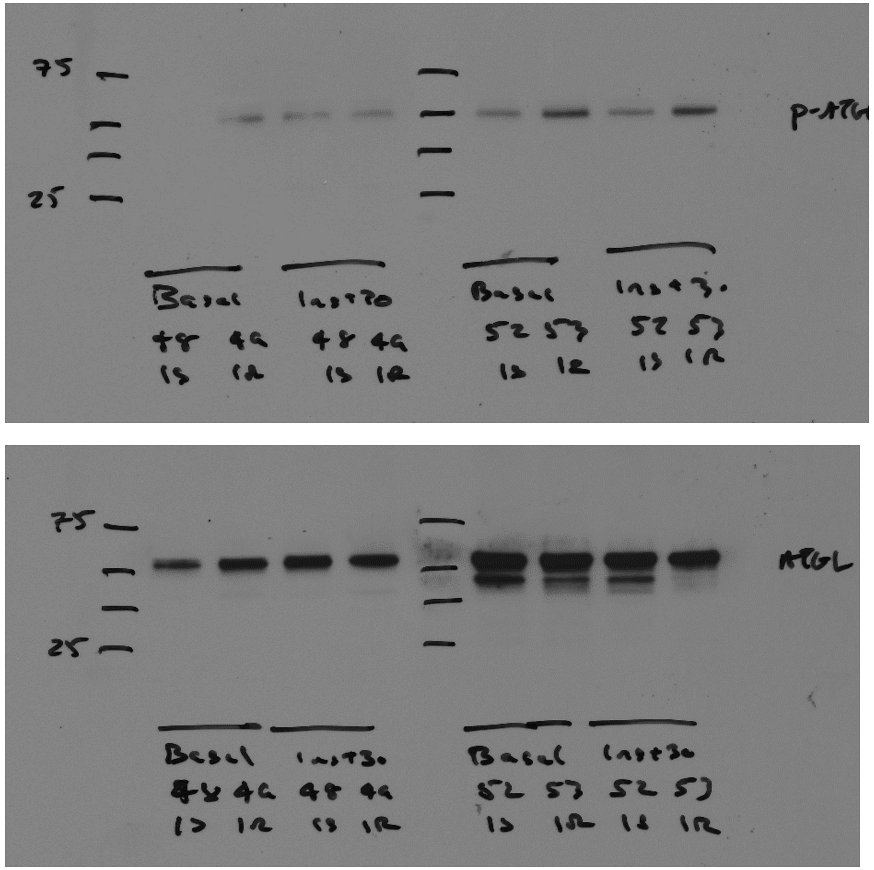

	Basal		Ins+30		Basal		Ins+30	
	IS	IR	IS	IR	IS	IR	IS	IR

D

p-HSL Ser^{600}

HSL

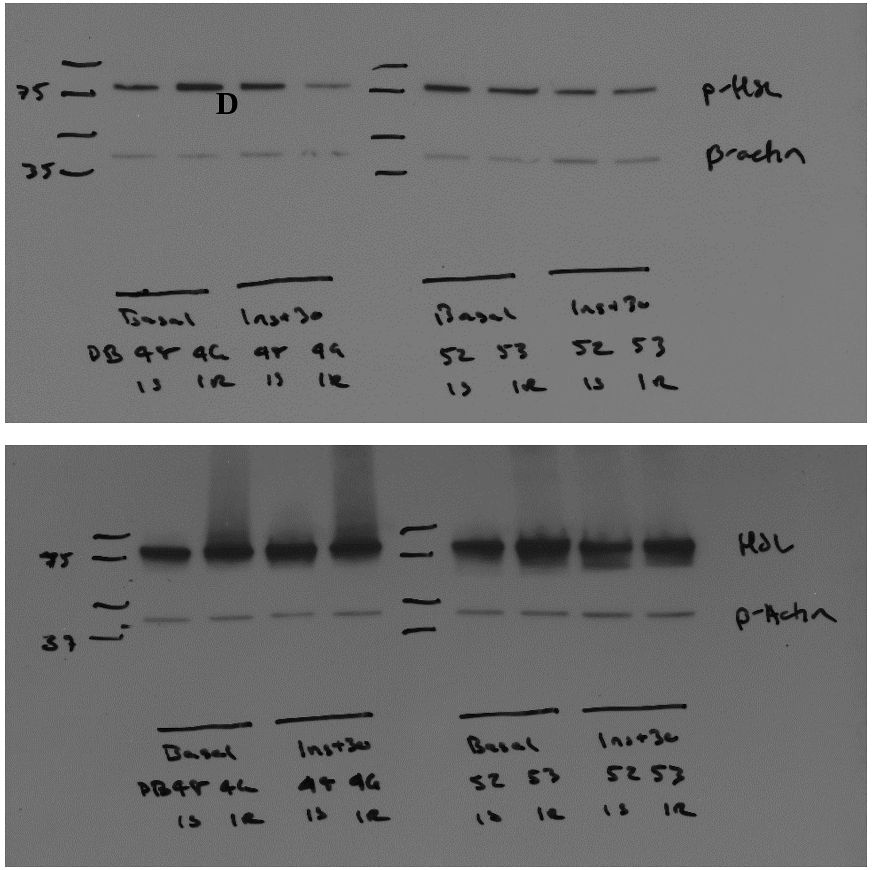

Figure 2. Unedited western blots images for the ratios of p-ATGL (Ser^{406})-to-ATGL and p-HSL (Ser^{660})-to-HSL prior to and in response to acute, low-dose insulin infusion (30-minutes following the initiation of 8 mU·m^{-2}·min^{-1} insulin). Image sets on the right were use as representative images.
